# Peroxiredoxin 5 protects HepG2 cells from ethyl β-carboline-3-carboxylate-induced cell death via ROS-dependent MAPK signalling pathways

**DOI:** 10.7150/jca.76663

**Published:** 2022-09-06

**Authors:** Dan-Ping Xie, Yi-Xi Gong, Jaihyung Lee, Eui Man Jeong, Chen-Xi Ren, Xiao-Yu Guo, Ying-Hao Han, Yu-Dong Cui, Seung-Jae Lee, Taeho Kwon, Hu-Nan Sun

**Affiliations:** 1 College of Life Science and Technology, Heilongjiang Bayi Agricultural University, Daqing, Heilongjiang 163319, P.R. China; 2 Jeju Research Institute of Pharmaceutical Sciences, College of Pharmacy, Jeju National University, Jeju, 63243, Republic of Korea; 3 Interdisciplinary Graduate Program in Advanced Convergence Technology and Science, Bio-Health Materials Core-Facility Center and Practical Translational Research Center, Jeju National University, Jeju, 63243, Republic of Korea; 4 Epigenetics Drug Discovery Center, Hwalmyeong Convalescence Hospital, Gapyeong, Gyeonggi 12458, Republic of Korea; 5 Functional Biomaterial Research Center, Korea Research Institute of Bioscience and Biotechnology, Jeongeup-si, Jeonbuk 56212, Republic of Korea; 6 Department of Applied Biological Engineering, Biotechnology of KRIBB School, University of Science and Technology, Daejeon 34113, Republic of Korea; 7 Primate Resources Center, Korea Research Institute of Bioscience and Biotechnology (KRIBB), Jeongeup-si, Jeonbuk, 56216, Republic of Korea; 8 Department of Functional Genomics, Bioscience of KRIBB School, University of Science and Technology, Daejeon 34113, Republic of Korea

**Keywords:** Peroxiredoxin5, ethyl β-carboline-3-carboxylate, ROS, mitochondria, HepG2 cells, liver cancer

## Abstract

Peroxiredoxin 5 (PRDX5) is the member of Prxs family, widely reported to be involved in various types of cell death. We previously found that *PRDX5* knockdown increases the susceptibility of cell death upon oxidative stress treatment. Ethyl β-carboline-3-carboxylate (β-CCE), an alkaloid extracted from *Picrasma quassioides*, has been reported to play a role in neuronal disease, but its anti-cancer potential on liver cancers remains unknown. Here, we studied the effect of PRDX5 on ethyl β-carboline-3-carboxylate (β-CCE)-induced apoptosis of hepatomas. High expression level of PRDX5 was deeply related with the postoperative survival of patients with liver cancer, indicating that PRDX5 may be a biomarker of live cancer processing. Moreover, PRDX5 over-expression in HepG2 cells significantly inhibited β-CCE-induced cell apoptosis and cellular ROS levels as well as mitochondrial dysfunction. Signalling pathway analysis showed that β-CCE could significantly up-regulate the ROS-dependent MAPK signalling, which were in turn boosts the mitochondria-dependent cell apoptosis. Moreover, PRDX5 over-expression could reverse the anti-cancer effects induced by β-CCE in HepG2 cells. Our findings suggest that PRDX5 has a protective role on β-CCE-induced liver cancer cell death and provides new insights for using its anti-cancer properties for liver cancer treatment.

## Introduction

Liver cancer is one of the six most dangerous and deadliest cancer forms in the world 1. Many factors, such as smoking, drinking, and disrupted sleep cycle contribute to the burden of liver cancer in today's society 2. Furthermore, despite advances in clinical treatment and diagnostics, multidisciplinary comprehensive treatment on a worldwide scale is required to improve liver cancer treatment efficacy, and researchers must continue to develop and apply new small molecule compounds 2.

β-carboline alkaloids extracted from natural herbs exhibit many pharmacological effects in medical treatment that differ based on the part of the plant and species from which they have been extracted 3. Ethyl β-carboline-3-carboxylate (β-CCE) belongs to a class of β-carboline alkaloids extracted from *Picrasma quassioides* 3. This kind of alkaloid has anti-inflammatory, anti-viral 4, anti-oxidative 5, anti-tumour, and calming effects on brain nerves 6. As one of the active constituents of β-carboline alkaloids, β-CCE has various medicinal properties. In the 19th century, β-CCE was certified as a benzodiazepine antagonist and involved in the regulation of nerve-related diseases 7. β-CCE acts as an antagonist of γ-aminobutyric acid receptors, and has the effect of stimulating spontaneous discharge in the hippocampus of rats and reducing the threshold and latency of convulsions induced 7. However, few studies have evaluated the anticancer properties of β-CCE.

Redox processes have always existed in cells, and reactive oxygen species (ROS) play vital roles in metabolic processes 8. ROS act as signalling molecules in biological systems, regulating signalling pathways 9, maintaining tissue homeostasis 10, and modulating cell survival and apoptosis 11. Within the normal physiological concentration range, ROS exert beneficial effects on cell growth and development by minimizing oxidative damage and satisfying energy demands 12. When the cell's tolerance range for high ROS is exceeded, it enters an oxidative stress condition. Various cellular antioxidants eliminate excess ROS in cells via redox processses to preserve essential physiological functioning 13. As the first line of defence against oxidation, peroxiredoxins (PRDXs) play a critical role in the fight against ROS 14. PRDX5, the fifth member of the PRDX family, is located in the cytoplasm and mitochondria of cells and is involved in anti-cancer and anti-inflammatory functions 15. We previously found that PRDX5 regulates ROS and nitric oxide generation in microglia, reducing lipopolysaccharide-induced activation 15. By modulating the Wnt/β-catenin signalling pathway, PRDX5 can govern the β-lapachone-induced apoptosis of SW480 colon cancer cells 16. It has also been discovered that mitochondrial PRDX5 regulates Ca^2+^ transport between the mitochondria and endoplasmic reticulum, thereby regulating mitochondria-dependent apoptosis 17.

In this study, we investigated the effect of PRDX5 on β-CCE-induced apoptosis of hepatoma cells. Our study fills a gap in anti-tumour research and provides a viable basis for liver cancer gene therapy and a novel theoretical basis for liver cancer chemotherapy.

## Materials and methods

### Cell lines

HepG2 (HB-8065), Hep3B (HB-8064), WRL68 (CL-48) cell lines were obtained from the American Type Culture Collection (ATCC; Rockville, MD, USA), whereas Huh-7 (CL-0120) and L-02 (CL-0111) cell lines were obtained from Procell (Procell Life Science & Technology Co., Ltd, Hubei, China). HepG2, Hep3B, and WRL68 cells were cultured in Eagle's Minimum Essential Medium (EMEM; Invitrogen, Carlsbad, CA, USA). Huh-7 cells were cultured in Dulbecco's Modified Eagle Medium (Invitrogen), while L-02 cell were cultured in Roswell Park Memorial Institute 1640 Medium (Invitrogen). All media were supplemented with 10% foetal bovine serum (FBS, Hyclone, Logan, UT, USA), 0.1 mg/mL streptomycin, and 100 units/mL penicillin (Solarbio life sciences, Beijing, China). All the cells were used after authentication by short tandem repeat-based DNA fingerprinting and multiplex polymerase chain reaction.

### Cell viability evaluation

The cells were seeded at a density of 5,000 cells/well in a 96-well plate (NEST Biotechnology, Wuxi, Jiangsu, China) and cultured at 37℃ with 5% CO_2_ for 24 h. Cell viability after treatment with β-CCE (Sigma-Aldrich, St. Louis, MO, USA) and other drugs was assessed using the 3-(4,5-dimethylthiazol-2-yl)-2,5-diphenyltetrazolium bromide (MTT; Multisciences, Jiangsu, China), colony formation assay, lactate dehydrogenase (LDH) release assay, and calcein-acetoxymethyl ester (AM) staining. Cell viability was also measured with or without N-acetyl-L-cysteine (NAC, Sigma-Aldrich) pre-treatment or fluorouracil (Solarbio life sciences) treatment. MTT assay was performed 2 h after β-CCE treatment, and a solubilisation buffer (dimethyl sulfoxide) was added for 15 min to solubilize the MTT formazan crystals. Absorbance was measured at 570 nm using UV MAX kinetic microplate reader (Molecular Devices, LLC, Sunnyvale, CA, USA). Colony formation assay was performed using crystal violet solution (Beijing Leagene Biotechnology Co., Ltd., Beijing, China), and the number of colonies cultured over 7 days was counted. In LDH release assay, an LDH releasing reagent was added to the cell culture plate for 1 h and the supernatant was recovered and incubated at room temperature for 30 min. Absorbance was subsequently measured at 490 and 600 nm using the UV MAX kinetic microplate reader (Molecular Devices). Cell death assay was performed by staining with calcein-AM (Sigma-Aldrich) and Hoechst 32258 (Thermo Fisher Scientific, Waltham, MA, USA). Nuclei were visualized by staining with Hoechst 32258 (Thermo Fisher Scientific) and observed qualitatively under a microscope after a 20-min incubation. Photomicrographs were taken using fluorescence microscope (EVOS^®^xl core cell culture microscope, Advanced Microscopy Group, Paisley, Scotland, UK). All assays were performed in triplicates.

### Western blotting

Cells plated in 6-well culture plates (NEST Biotechnology) were grown till approximately 70% confluency and subsequently subjected to β-CCE treatment with or without NAC. Cells were lysed on ice using lysis buffer. A total of 20 μg protein was resolved by 12% sodium dodecyl sulfate-polyacrylamide gel electrophoresis, transferred onto nitrocellulose membranes (Millipore, Bedford, MA, USA), and probed with primary antibody overnight at 4℃. The membranes were washed five times with tris-buffered saline containing Tween-20 (GenStar, Beijing, China) and incubated with secondary antibodies for 1 h at room temperature. The following primary antibodies were used: Bcl-2, Bcl-xL, Bad, Bax, Cleaved Caspase-3, Cleaved Caspase-7, Cleaved Caspase-9 (Cell Signaling Technology, Beverly, MA, USA; dilution, 1:1000), p-ERK, p-JNK, p-P38, ERK, JNK, P38 (Bioss, Beijing, China; dilution, 1:500), PRDX1, PRDX2, PRDX3, PRDX4, PRDX5, and PRDX6 (AbFrontier, Seoul, South Korea; dilution, 1:2000). β-actin (Abcam, Cambridge, MA, USA; dilution, 1:5000) was used as loading control. The following secondary antibodies were used: horseradish peroxidase-conjugated goat anti-rabbit IgG or anti-mouse IgG (Sangon Biotech, Shanghai, China). Working solutions of the ECL Western Blotting Detection Kit (Vazyme Biotech Co., Ltd, Nanjing, China) and chemiluminescence detection system (GE Healthcare Life Sciences, Chalfont, UK) were used according to the manufacturer's protocol to detect and visualize the proteins.

### ROS production measurements

ROS generation was measured using dihydroethidium (DHE; Beyotime Biotechnology, Shanghai, P.R. China) exposure for 30 min at 37 °C in the supernatant of differently treated cells. Mitochondrial superoxide generation was measured using MitoSOX (Thermo Fisher Scientific). Nuclei were visualized using the Hoechst dye. The stained cells were observed and photographed under a fluorescent microscope (EVOS^®^xl core cell culture microscope).

### Mitochondrial depolarization assay

Changes in cell mitochondrial membrane potential (MMP) were evaluated by staining with 20 mM JC-1 (Beyotime, Shanghai, PR China) for 30 min at 37℃ in the supernatant of differently treated cells. Stained cells were observed and photographed under a fluorescent microscope (EVOS^®^xl core cell culture microscope).

### Gene expression analysis

*PRDX5* expression levels in normal or tumour tissue samples of patients with hepatocellular carcinoma (HCC) were analysed using the TNMplot online database (https://tnmplot.com/analysis/), a web tool used for comparing gene expression in normal, tumour, and metastatic tissues 18. Protein expression was analysed online using the Human Protein Atlas (https://www.proteinatlas.org/) database 19. The correlation between PRDX5 and survival rate in patients with HCC was evaluated online using the Kaplan-Meier plotter (http://kmplot.com/analysis/index.php) database, an online system to analyse mRNA Affymetrix Genechip and RNA-sequencing datasets for patients with HCC. All gene expression data used in this study was secondary data obtained from online databases; no primary data was generated.

### Construction of cells with stable PRDX5 over-expression

His-tagged PRDX5 LV3 (H1&Puro) and control RNA LV3 (H1&Puro) lentiviral vectors were purchased from Shanghai GenePharma Co., Ltd. (Shanghai, China). HepG2 cells were seeded in a 6-well culture plate (NEST Biotechnology) at a density of 2 × 10^5^/well and incubated for 24 h (37 °C and 5% CO_2_) prior to infection. The culture medium was replaced with polybrene (5 μg/ml; Shanghai GenePharma Co., Ltd.) and packed lentivirus were added at a multiplicity of infection of 20 for 24 h, and subsequently replaced with complete culture medium (EMEM with 10% FBS and antibiotics). Infected cells were selected by treatment with 10 μg/mL G418 (Solarbio life sciences). *PRDX5* expression level was examined by western blotting.

### Statistical analysis

All statistical analyses were performed using Graphpad Prism software (Version 8.0; San Diego, CA, USA), and data are presented as means ± standard deviations from at least three independent experiments. One-way analysis of variance and Tukey's post-hoc test were performed to analyse the significance of differences among groups. P values < 0.05 were considered statistically significant.

## Results

### Cytotoxic effect of β-CCE on hepatoma cells

**Figure 1:**
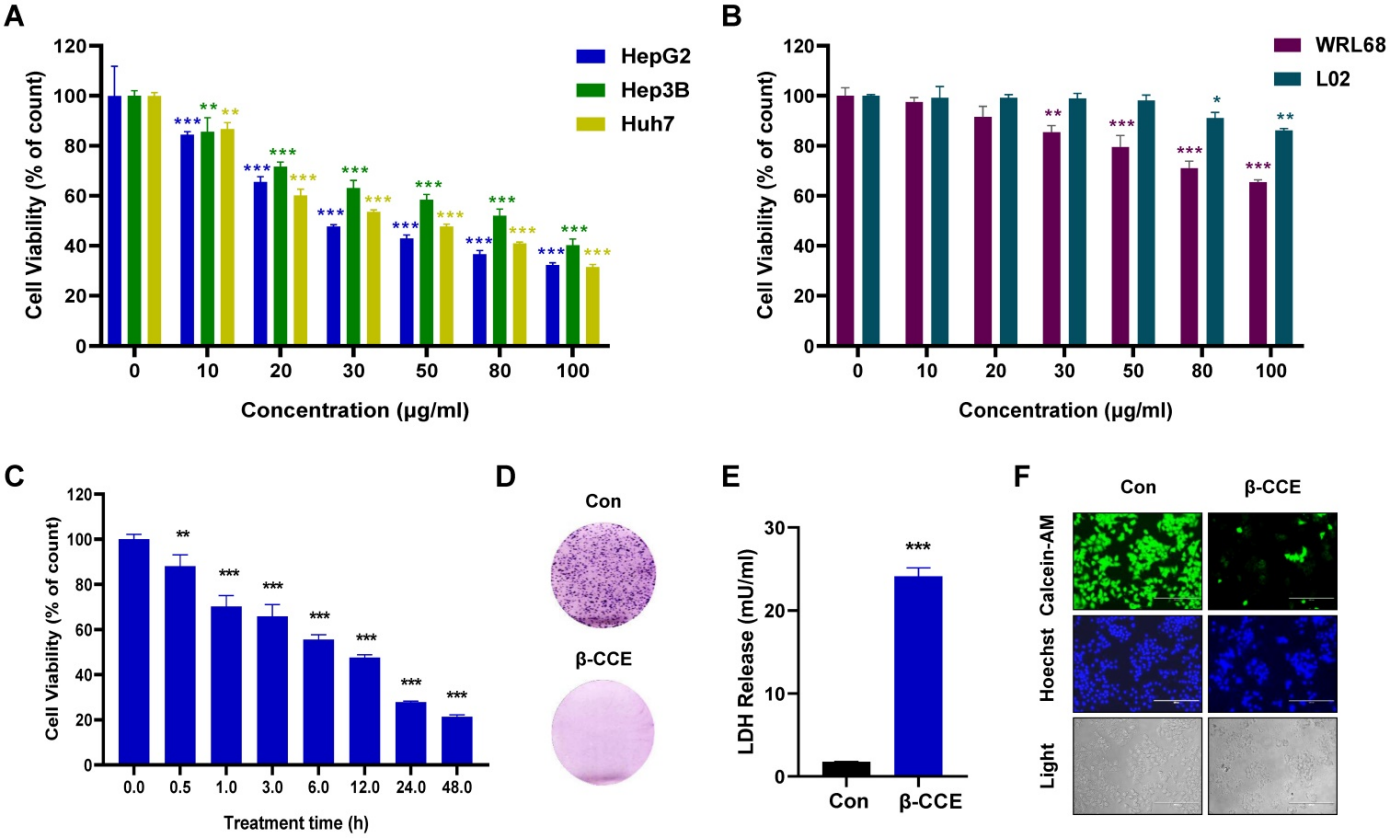
** Cytotoxicity of β-CCE on hepatoma cells. (A)** Effect of varying concentrations of β-CCE (0, 10, 20, 30, 50, 80, and 100 μg/mL) on the viability of HepG2, Hep3B, and Huh7 cells detected by MTT Assay. **(B)** Effect of varying concentrations of β-CCE (0, 10, 20, 30, 50, 80, and 100 μg/mL) on the viability of WRL68 and L02 cells detected by MTT Assay. **(C)** Cell viability of HepG2 cells treated with 80 μg/mL β-CCE for different time periods (0, 0.5, 1, 3, 6, 12, 24 h) determined by MTT assay. **(D)** Effect of β-CCE on the colony formation ability of HepG2 cells examined by fluorescence microscopy (scale bar = 100 μM). **(E)** β-CCE at concentrations of 80 µg/mL induced HepG2 cell injury as evaluated by LDH assay. **(F)** HepG2 cells were treated with β-CCE at a concentration of 80 μg/mL for 24 h. Calcein-AM staining was used to detect the viability of HepG2 cells. Calcein-AM (green), Hoechst (blue), and light images were measured by fluorescence microscopy (scale bar = 400 μM).

To evaluate β-CCE cytotoxicity, hepatoma cells (Huh7, Hep3B, and HepG2) treated with different concentrations (0, 10, 20, 30, 50, 80, 100 μg/ml) of β-CCE for 24 h were subjected to MTT assay. As shown in Figure 1A, β-CCE had perceptible cytotoxic effects; it induced death in a significant number of hepatoma cells, especially in HepG2 cells. Concurrently, it was observed that β-CCE was less toxic to non-tumour cell lines (WRL68 and L02 cell lines) (Figure 1B). Furthermore, on treating HepG2 cells with 80 μg/mL β-CCE for different time periods (0, 0.5, 1, 3, 5, 12, 24, and 48 h), we observed that β-CCE exhibited an inhibitory effect on HepG2 cell survival rate when treated even for a short time period; HepG2 cells showed a time-dependent inhibition on β-CCE treatment (Fig. 1C). β-CCE treatment also inhibited HepG2 colony formation ability (Fig. 1D) and increased LDH release level (Fig. 1E). Next, we detected the changes of calcein-AM by fluorescence microscopy, which was used to directly reflect the number and status of living cells, whereas Hoechst was used to simultaneously stain the nuclei. As depicted in Fig. 1F, the number of viable HepG2 cells was significantly reduced after their treatment with 80 µg/mL β-CCE for 24 h. Nuclear chromatin fluorescence intensity of the treated group was significantly higher than that of the untreated group; cell shrinkage was also evidently observed under the light field of view. This indicates that β-CCE inhibits the survival of HepG2 cells through apoptosis and may be a small molecule drug worthy of research and development in the treatment of hepatoma cells.

### β-CCE induces mitochondria-dependent apoptosis in HepG2 cells

**Figure 2:**
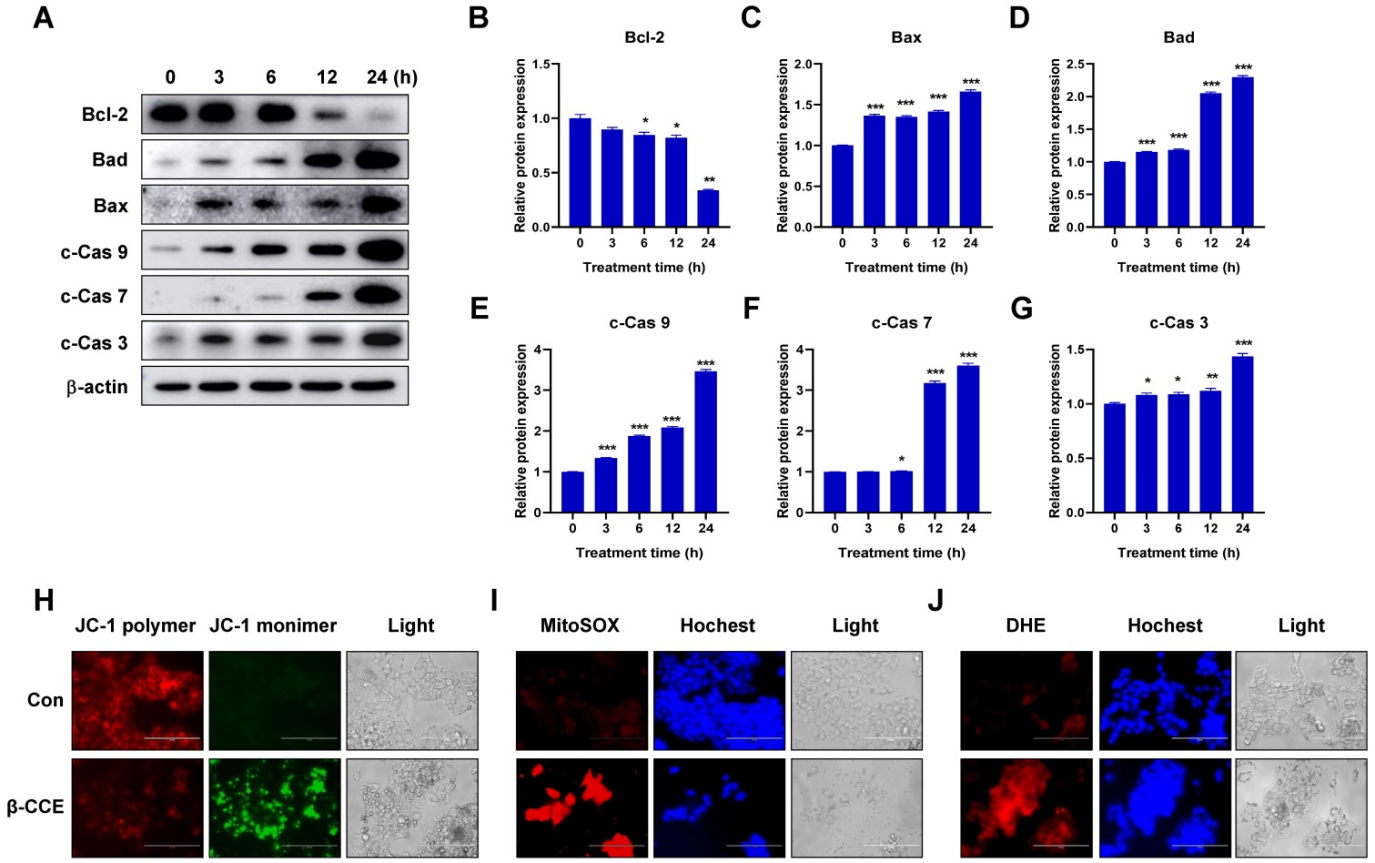
** β-CCE induced apoptosis-related protein changes, mitochondrial dysfunction, and ROS accumulation in HepG2 cells. (A)** Bcl-2, Bad, Bax c-Cas 9, c-Cas 7, and c-Cas 3 protein expression levels in HepG2 cells after treatment with 80 µg/mL β-CCE for different time periods (0, 3, 6, 12, and 24 h). **(B-G)** Quantitative analysis of (B) Bcl-2, (C) Bax, and (D) Bad (E) c-Cas 9, (F) c-Cas 7 and (G) c-Cas 3 expression levels in HepG2 cells after treatment with 80 µg/mL β-CCE for different time periods (0, 3, 6, 12, and 24 h). Data are presented as standard error of the mean of three different samples (*p < 0.05, **p < 0.01, ***p < 0.001). **(H)** JC-1 staining was performed to detect changes in MMP in HepG2 cells after treatment with 80 µg/mL β-CCE for 24 h. Changes in MMP were observed as weakening red fluorescence. JC-1 polymer (red), JC-1 monomer (green), and light images were measured by fluorescence microscopy (scale bar = 400 µM). **(I)** MitoSOX staining was performed to detect β-CCE (80 µg/mL) effect on mitochondrial ROS levels in HepG2 cells. MitoSOX (red), Hoechst (blue), and light images were measured by fluorescence microscopy (scale bar = 400 µM). **(J)** DHE staining was used to detect β-CCE (80 µg/mL) effects on intracellular ROS levels in HepG2 cells. DHE (red), Hoechst (blue), and light images were measured by fluorescence microscopy (scale bar = 400 µM).

To further explore the mechanism through which β-CCE induces cell apoptosis, we first performed western blotting to detect changes in the levels of Bcl-2 family-related proteins, which are regulators of apoptosis. The expression of anti-apoptotic protein Bcl-2 in HepG2 cells was significantly decreased with prolonged exposure to β-CCE (0, 3, 6, 12, and 24 h) (Fig. 2A and 2B). In contrast, pro-apoptotic proteins Bad (Fig. 2C) and Bax (Fig. 2D) were significantly increased on prolonged exposure of HepG2 cells to β-CCE, indicating that β-CCE induces apoptosis in HepG2 cells by altering the expression of Bcl2 family proteins. Next, we examined changes in the levels of c-Cas3, c-Cas7, and c-Cas9, which are related to mitochondrial apoptosis pathway. As can be perceived from Fig. 2A, the levels of c-Cas9 (Fig. 2E), c-Cas7 (Fig. 2F), and c-Cas3 (Fig. 2G) were significantly increased on prolonged exposure to β-CCE (0, 3, 6, 12, and 24 h). Subsequently, we detected changes in MMP in the control group and β-CCE-treated group cells by fluorescence photomicrography (Fig. 2H). We found that JC-1 polymer was significantly reduced and JC-1 monomer was significantly increased after 24 h of β-CCE treatment. This suggests that, following β-CCE treatment, HepG2 cells undergo depolarization, resulting in a decrease in intracellular MMP. Simultaneously, we also detected ROS levels in the mitochondria (Fig. 2I) and cytoplasm (Fig. 2J) and observed significantly increased ROS levels in both mitochondria and cytoplasm after 24 h of β-CCE treatment. Therefore, these data suggest that β-CCE causes mitochondrial dysfunction by reducing the intracellular MMP and induces apoptosis by increasing intracellular and mitochondrial ROS levels.

### Effect of ROS on β-CCE-induced HepG2 cell apoptosis

**Figure 3:**
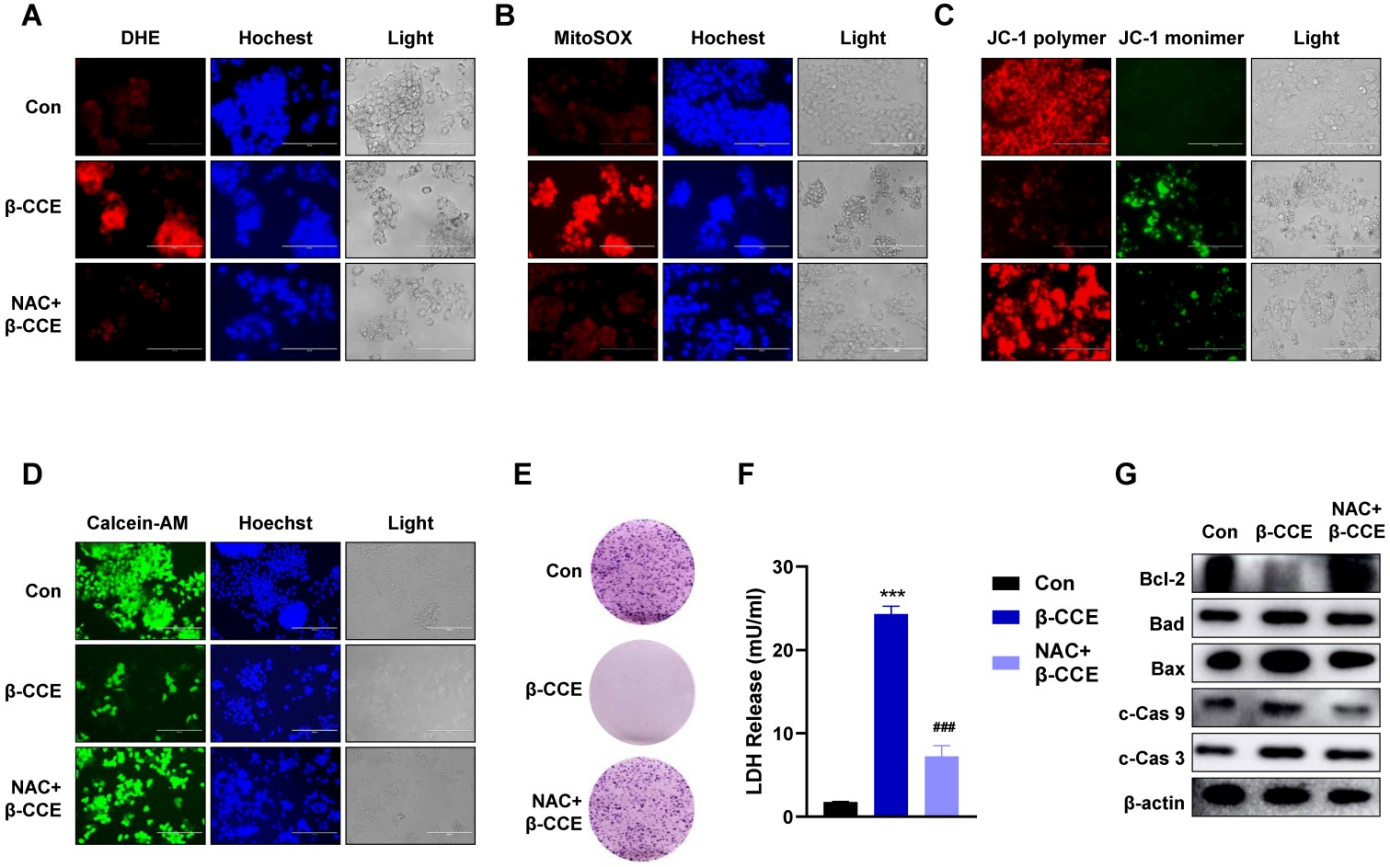
** Effect of intracellular ROS inhibition on colony forming ability and apoptosis-related protein expression in HepG2 cells.** HepG2 cells were pre-treated with NAC for 30 min and subsequently treated with β-CCE (80 µg/mL) for 24 h. The three treatment groups included control (Con), β-CCE treatment (β-CCE), NAC and β-CCE co-treatment (NAC+β-CCE) groups. **(A)** DHE staining was performed to detect the effect of intracellular ROS levels in HepG2 cells of each treatment group. DHE (red), Hochest (blue), and light images were measured by fluorescence microscopy (scale bar = 400 µM). **(B)** MitoSOX staining was performed to evaluate the effect of mitochondrial ROS levels in HepG2 cells of each treatment group. MitoSOX (red), Hochest (blue), and light images were measured by fluorescence microscopy (scale bar = 400 µM). **(C)** JC-1 staining was performed to examine changes in the MMP of HepG2 cells in each treatment group. JC-1 polymer (red), JC-1 monomer (green), and light images were measured by fluorescence microscopy (scale bar = 400 µM). **(D)** Calcein-AM staining was performed to detect the viability of HepG2 cells in each treatment group. Calcein-A (green), Hoechst (blue), and light images were measured by fluorescence microscopy (scale bar = 400 µM). **(E)** HepG2 cell proliferation was measured by colony formation assay (scale bar = 100 µM). **(F)** HepG2 cell injury in each group was assessed by LDH release assay (***p < 0.001, ^###^p < 0.001). **(G)** Western blotting was performed for detecting the expression of apoptosis-related proteins, such as Bcl-2, Bad, Bax, c-Cas 9, and c-Cas 3 in HepG2 cells of each treatment group.

To explore whether ROS induced by β-CCE treatment is vital for HepG2 cell apoptosis, HepG2 cells were pre-treated with the ROS scavenger NAC. On detecting intracellular and mitochondrial ROS levels, it was found that intracellular and mitochondrial ROS levels decreased significantly after the removal of ROS generated by β-CCE (Fig. 3A and 3B). Furthermore, MMP was restored (Fig. 3C), and the number of viable cells in the visual field increased significantly (Fig. 3D). The colony forming ability of HepG2 cells was also altered (Fig. 3E), and LDH release was also alleviated (Fig. 3F). Subsequently, we detected changes in the expression of apoptosis-related proteins. As depicted in Fig. 3G, after pre-treatment with NAC, the expression levels of anti-apoptotic protein Bcl-2 as well as pro-apoptotic proteins Bad and Bax were increased. In contrast, c-Cas9 and c-Cas3 expression levels were also reduced, which proved that β-CCE-induced apoptosis of HepG2 cells was significantly reduced after pre-treatment with NAC. This suggests that β-CCE-induced increase in apoptosis can be reversed by ROS inhibition.

### Role of PRDX family in β-CCE-induced HepG2 cell apoptosis

**Figure 4:**
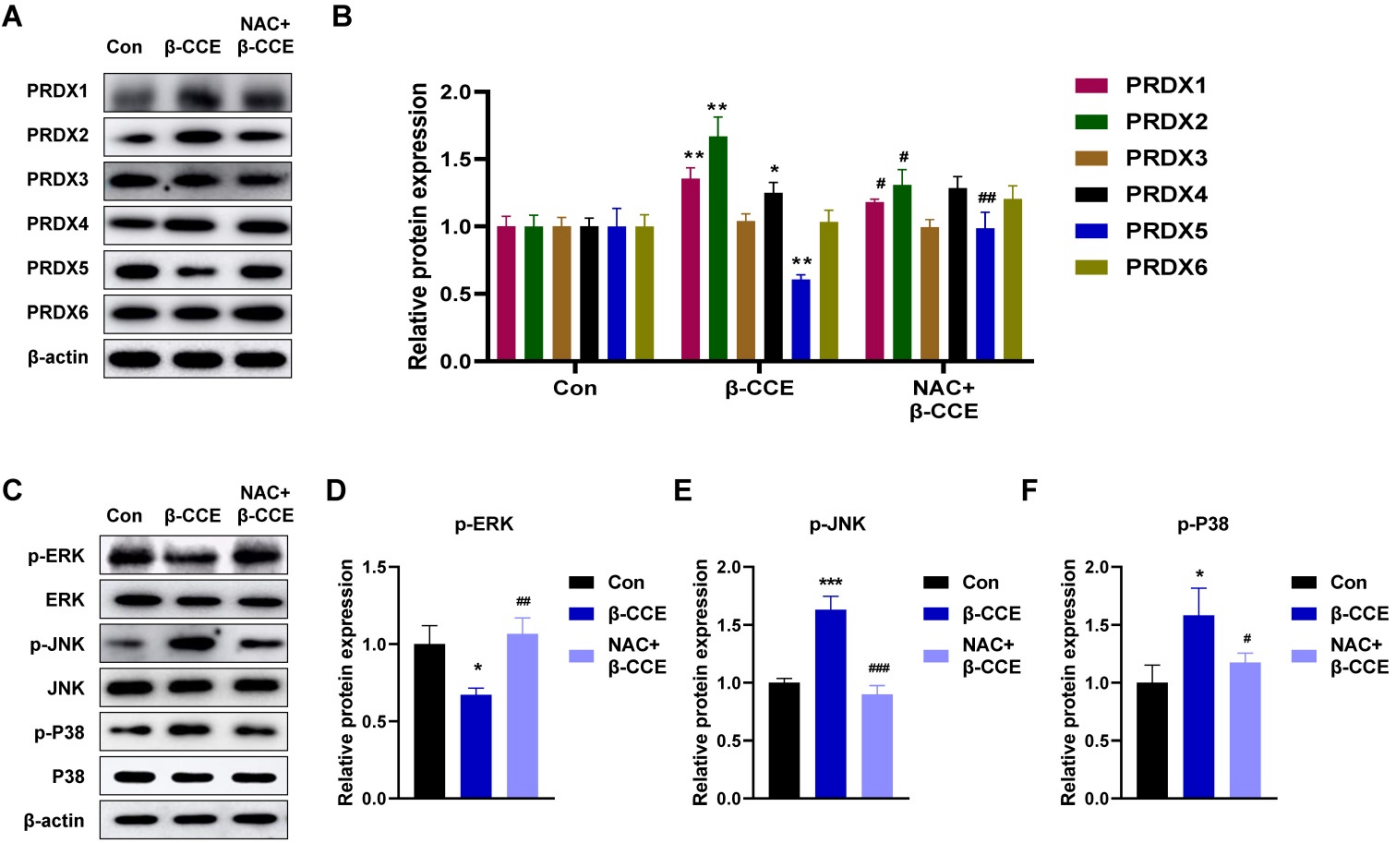
** Effects of β-CCE on PRDX family proteins and MAPK signalling pathway in HepG2 cells.** HepG2 cells were pre-treated with NAC for 30 min and subsequently treated with β-CCE (80 μg/mL) for 24 h. **(A)** The expression of PRDX family proteins in HepG2 cells of each treatment group (Con, β-CCE, and NAC+β-CCE) was detected by western blotting. **(B)** Quantitative analysis of PRDX (I-VI) protein expression level. **(C)** The expression of ERK, JNK, P38, and their phosphorylated proteins (p-ERK, p-JNK, and p-P38) in HepG2 cells of each treatment group (Con, β-CCE, NAC+β-CCE) was detected by western blotting. **(D-F)** Quantitative analysis of (D) p-ERK, (E) p-JNK, and (F) p-P38 expression levels. *** represents the difference between β-CCE and Con groups, ### represents the difference between β-CCE and NAC+β-CCE groups (**p < 0.01, ***p < 0.001, ^###^p < 0.001).

Next, we examined any changes in the expression levels of PRDX family proteins. As major scavengers of ROS, PRDX1 and PRDX2 quickly scavenged ROS caused by foreign substances, showing an upward trend. PRDX5 expression level was observed to decrease; however, it was restored to a level similar to that in the untreated group after NAC pre-treatment (Fig. 4A and 4B). After β-CCE treatment, the MAPK signalling pathway was significantly activated (Fig. 4C), p-ERK expression level was significantly decreased (Fig. 4D), and p-JNK and p-P38 expression levels were significantly increased. This suggests that β-CCE induces apoptosis through the MAPK signalling pathway and possibly by depleting PRDX5.

### PRDX5 as a biomarker for liver cancer

**Figure 5:**
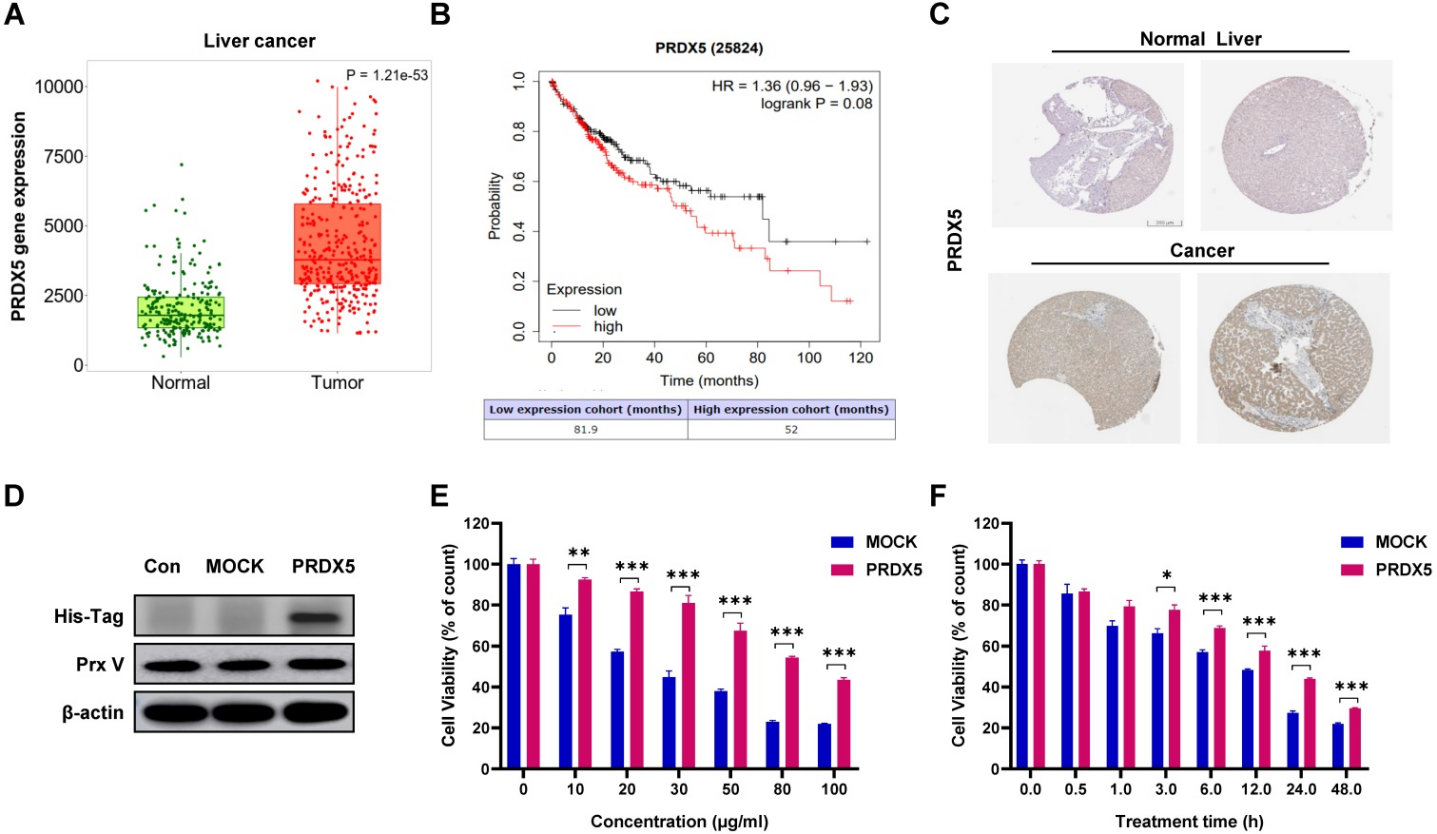
** Effects of PRDX5 on the development and progression of HCC. (A)** *PRDX5* expression levels in tumour and normal liver tissue from patients with HCC (green box represents normal liver tissues; red box represents tumour tissues). **(B)** Survival analysis based on *PRDX5* expression levels in liver tissues of patients (black line represents patients with low PRDX5 expression in liver tissues; red line represents patients with high PRDX5 expression in liver tissues). **(C)** Difference in PRDX5 expression in normal liver tissue and HCC tissue were analysed using pathological tissue sections. **(D)** Identification of PRDX5-overexpressing HepG2 cell line by western blotting (PRDX5 transfection sequence with His-Tag). **(E)** Effect of varying β-CCE concentrations (0, 10, 20, 30, 50, 80, and 100 μg/mL) on the viability of MOCK and PRDX5-over-expressing HepG2 cells was evaluated by MTT assay. **(F)** Viability of MOCK and PRDX5-over-expressing HepG2 cells treated with 80 μg/mL β-CCE for different time periods (0, 0.5, 1, 3, 6, 12, and 24 h) evaluated by MTT assay (*p < 0.05, **p < 0.01, ***p < 0.001).

Using data from bioinformatics databases, we analysed the effect of PRDX5 expression on HCC and compared postoperative survival between different liver cancer patients and normal patients. In addition, *PRDX5* mRNA expression was observed to be incrementally up-regulated with normal and tumour tissues analysed in the TNMplot database (p = 1.21e-53) (Fig. 5A). In Kaplan-Meier plotter analysis, liver cancer patients with higher *PRDX5* expression had worse overall survival than those with lower *PRDX5* expression (Fig. 5B). The survival time of patients with low *PRDX5* expression in liver tissue (81.9 months) was significantly longer than that of patients with high PRDX5 expression (52 months). Moreover, immunohistochemistry data for liver cancer using the Human Protein Atlas revealed that PRDX5 protein was moderately expressed in normal liver tissues but abundantly expressed in liver cancer tissues (Fig. 5C). These data indicate the critical roles of PRDX5 in the progression of liver cancer. To explore the effects caused by the difference in PRDX5 content in liver cancer tissue on β-CCE-induced cell death, we used lentiviral vectors to over-express the PRDX5 gene in HepG2 cells (Fig. 5D). MTT assay was performed to detect changes in the viability of HepG2 cells treated with various concentrations of β-CCE for different time periods. The resistance of HepG2 cells to β-CCE treatment and their survival rate was significantly increased on over-expression of PRDX5 (Fig. 5E and 5F). This suggests that PRDX5 regulates β-CCE-induced apoptosis in hepatoma cells.

### PRDX5 over-expression causes resistance to β-CCE-induced apoptosis via MAPK signalling pathway

**Figure 6:**
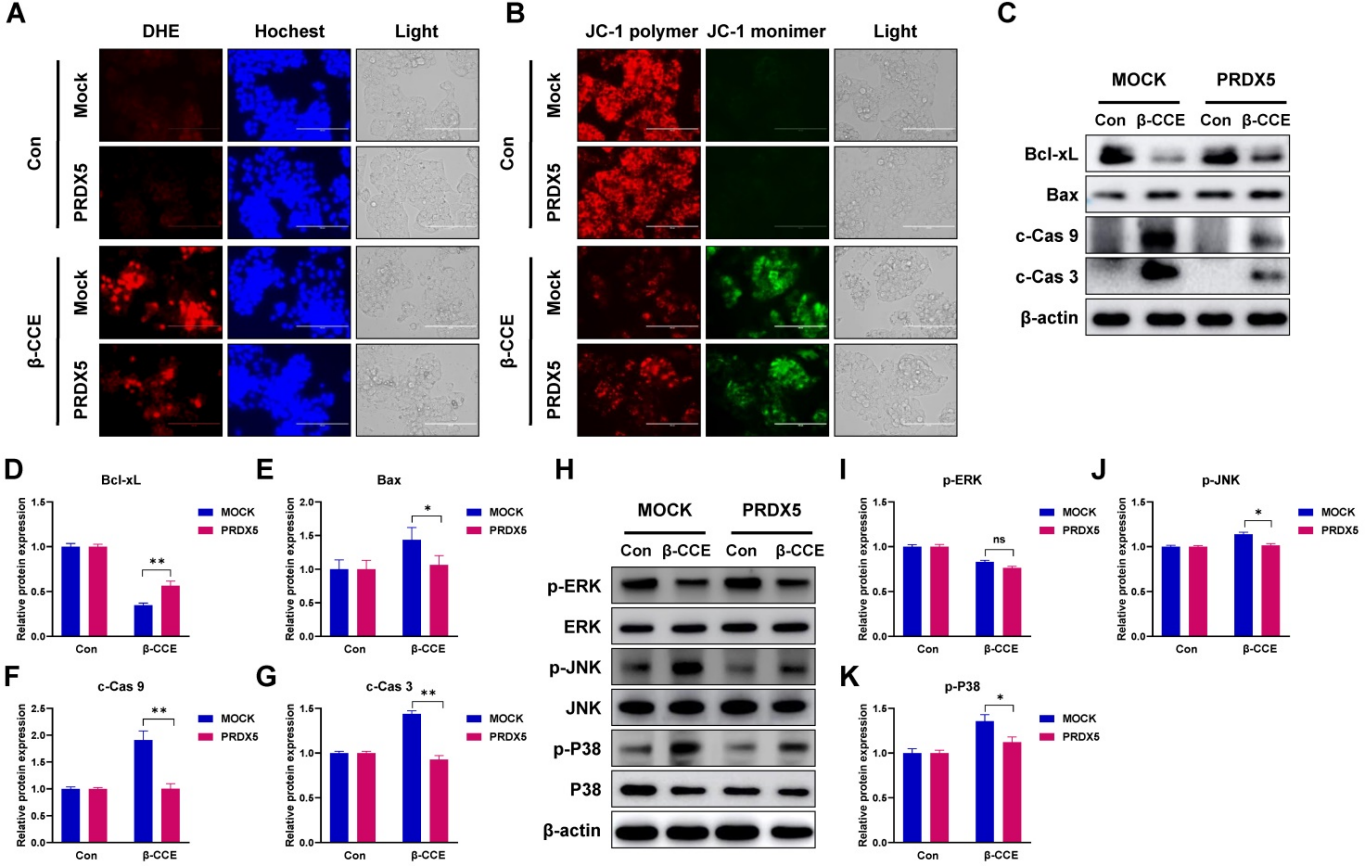
** Effects of PRDX5 over-expression on β-CCE-induced intracellular ROS and apoptosis-related protein expression levels and MAPK signalling pathway. (A)** Changes in the levels of intracellular ROS in MOCK and PRDX5-over-expressing HepG2 cells treated with β-CCE (80 µg/mL) for 24 h were detected by DHE staining. DHE (red), Hoechst (blue), and light images were measured by fluorescence microscopy (scale bar = 400 µM). **(B)** Changes in the MMP of MOCK and PRDX5-over-expressing HepG2 cells treated with β-CCE (80 µg/mL) for 24 h were detected by DHE staining. JC-1 polymer (red), JC-1 monomer (green), and light images were measured by fluorescence microscopy (scale bar = 400 µM). **(C)** The expression levels of apoptosis-related proteins in MOCK and PRDX5-over-expressing HepG2 cells treated with β-CCE (80 µg/mL) for 24 h were detected by western blotting. **(D-G)** Quantitative analysis of (D) Bcl-xL, (E) Bax, (F) c-Cas 9, and (G) c-Cas 3 expression levels. **(H)** The expression of ERK, JNK, and P38 and their phosphorylated proteins (p-ERK, p-JNK, and p-P38) in MOCK and PRDX5-over-expressing HepG2 cells treated with β-CCE (80 µg/mL) for 24 h were detected by western blotting. **(I-K)** Quantitative analysis of (I) p-ERK, (J) p-JNK, and (K) p-P38 expression levels (*p < 0.05, **p < 0.01).

One of the most important roles of PRDX5 is to regulate intracellular oxidative balance. Hence, we evaluated changes in ROS levels and MMP in PRDX5-over-expressing HepG2 cells. Intriguingly, PRDX5 over-expression did not affect ROS levels (Fig. 6A) or alter MMP (Fig. 6B) in untreated cells but enhanced β-CCE-induced changes in treated cells. Next, we detected the levels of Bcl-2 family proteins, c-Cas9, and c-Cas3 by western blotting (Fig. 6C), and observed that Bcl-xL (Fig. 6D) expression level was increased in β-CCE-induced PRDX5-over-expressing cells, whereas Bax, c-Cas9, and c-Cas3 (Fig. 6E-G) expression levels were decreased in β-CCE induced PRDX5-over-expressing cells. Furthermore, we investigated changes in the MAPK signalling pathway by western blotting. As illustrated in Fig. 6H, PRDX5 over-expression can effectively reduce the phosphorylation of P38 and JNK caused by β-CCE. This indicates that PRDX5 regulates β-CCE-induced apoptosis through P38 and JNK/MAPK signalling pathways.

## Discussion

HCC is one of the most prevalent malignant tumors worldwide 1. Despite advances in medical technology, the incidence of liver cancer continues to rise every year. Preventing and treating liver cancer is still a major hurdle for humans in the 21st century 1. In the ongoing process of drug development, chemotherapy medications have gradually become the first choice of treatment. These chemotherapeutic drugs exhibit anti-cancer effects such as regulating intracellular ROS levels by generating DNA damage in cancer cells 20, initiating cell cycle organization 21, and creating mitochondrial dysfunction 22.

ROS is the main cause of oxidative stress, and the basal level of redox processes in cancer cells is relatively high 23. Therefore, an increase in ROS levels can promote cell proliferation, leading to genetic instability and cancer occurrence and progression. Therefore, it is crucial to target different types of cancers by inducing ROS production leading to mitochondrial dysfunction. In this study, we evaluated the effect of β-CCE and discovered that it exhibits significant cytotoxicity against HepG2 cells in a time- and concentration-dependent manner and a low cytotoxicity against normal hepatocytes. When cells are stimulated by the environment, the relevant signalling pathways and antioxidant mechanisms in the cells are activated. The mitochondrial membrane is the primary target of oxidative damage. Mitochondria are like a factory for biological processes, with key roles in energy metabolism 24, redox balance, and apoptosis regulation 25.

β-CCE induces apoptosis in HepG2 cells via a mitochondria-dependent mechanism. The pro-apoptotic protein Bax is transferred from the cytoplasm to the mitochondria and binds to the mitochondrial membrane, resulting in the permeabilization of mitochondrial membrane and release of cytochrome c, SMAC, and other proteins from the mitochondrial intermembrane space into the cytoplasm, which continues to activate downstream Caspase9 26. Simultaneously, the active Caspase9 acts as an initiator Caspase, cleaving and activating the downstream effector Caspase3 and 7, resulting in the formation of an apoptotic cascade 27. JC-1 fluorescence labelling revealed that β-CCE can cause mitochondrial damage by reducing MMP and altering membrane permeability. To verify whether β-CCE-induced apoptosis in HepG2 cells was caused by a large accumulation of ROS, NAC was used to inhibit intracellular ROS. It was found that the MMP was significantly increased. To balance the accumulation of excess ROS generated in cells, there are antioxidant balance mechanisms in cells and organisms that can effectively suppress the production of intracellular free radicals, making ROS attacks on other intracellular organelles and even other substances in the body more vulnerable.

The PRDX family has a central role in the antioxidant process; it acts as a regulator of ROS concentration in many mammals in response to various intracellular signalling pathways to regulate various physio-pathological activities of cells 28. We performed western blotting to detect changes in the expression levels of various PRDX family proteins and discovered that the expressions of PRDX1, PRDX2, and PRDX5 were dramatically altered after treatment of HepG2 cells with β-CCE. Therefore, we focussed on PRDX5 in latter studies in our research. PRDX5 is an important antioxidant that exhibits strong antioxidant capacities in cellular mitochondria and cytoplasm to maintain intracellular redox balance 29. After performing a genetic analysis of patients with liver cancer, we found higher PRDX5 expression in the liver tissue of patients with liver cancer than in normal liver tissues. Concurrently, high PRDX5 levels also negatively correlated with patient survival after recovery. We over-expressed PRDX5 in HepG2 cells to explore whether a higher level of PRDX5 in liver cancer patients is related with chemotherapy resistance. Through cell viability assays, we discovered that PRDX5 over-expression reduced β-CCE-induced cytotoxicity to cells. This suggests that PRDX5 has a protective effect against drug treatment in HepG2 cells. We hypothesized that PRDX5 promotes cancer by targeting the levels of ROS because the expression of PRDX5 and the content of ROS generation in HepG2 cells were inversely correlated. By comparing the intracellular ROS generation levels between MOCK and PRDX5-over-expressing cells, we discovered that ROS level of PRDX5-over-expressing cells was repressed, and the stress of MMP was proportionally lowered following β-CCE treatment of cells. PRDX5 protects HepG2 cells from β-CCE-induced cytotoxicity by regulating ROS levels in HepG2 cells. PRDX5 over-expression in HepG2 cells decreased their sensitivity to β-CCE as well as the degree of intracellular ROS and mitochondrial damage. Although mitochondria's functional qualities in this process may make them a target, their abnormalities may not induce normal cell death in this process. Therefore, the difference between mitochondria in normal cells and cancer cells should be targeted for treatment, either by depleting antioxidants and increasing ROS production to cause oxidative damage to cells and apoptosis or by depleting antioxidants and increasing ROS production to cause oxidative damage to cells and apoptosis. However, our study has a limitation; we did not demonstrate the role of β-CCE and PRDX5 *in vivo*, in an animal model.

Our results suggest that β-CCE induces mitochondrial damage in cells by increasing the accumulation of intracellular ROS and activating the MAPK signalling pathway to induce apoptosis in HepG2 cells. Concurrently, after ROS scavenging, the apoptotic effect was significantly weakened. In this process, PRDX5 also plays an inhibitory role in regulating intracellular ROS level, which further provides an effective diagnosis and treatment mechanism for PRDX5 in the case of liver cancer. By increasing the intracellular ROS concentration to induce apoptosis, it provides a solution for the generation of drug resistance during the treatment of liver cancer. This will aid in eliminating resistance to chemotherapeutic drugs and enable cancer treatment.
